# Identification and Characterization of Key Chemical Constituents in Processed *Gastrodia elata* Using UHPLC-MS/MS and Chemometric Methods

**DOI:** 10.1155/2019/4396201

**Published:** 2019-10-23

**Authors:** Xide Ye, Yanhong Wang, Jianping Zhao, Mei Wang, Bharathi Avula, Qiaozhen Peng, Hui Ouyang, Zhong Lingyun, Jinlian Zhang, Ikhlas A. Khan

**Affiliations:** ^1^School of Pharmacy, Jiangxi University of Traditional Chinese Medicine, Nanchang, Jiangxi 344000, China; ^2^National Center for Natural Products Research, School of Pharmacy, The University of Mississippi, Jackson, MS 38677, USA; ^3^School of Computer Information Engineering, Nanchang Hangkong University, Nanchang, Jiangxi 330063, China; ^4^Division of Pharmacognosy, Department of BioMolecular Sciences, School of Pharmacy, The University of Mississippi, Jackson, MS 38677, USA

## Abstract

*Gastrodia elata* Blume belongs to the Orchidaceae family. *G. elata* is often processed when used in traditional Chinese medicine (TCM). In the current study, a traditional processing method, known as “Jianchang Bang,” was applied. Steamed and dried (S&D) *G. elata* was processed with ginger juice for up to 5 days (GEP5D). An UHPLC-MS/MS combined with a chemometric method was developed for the analysis of processed *G. elata* along with the raw material as well as steamed and dried *G. elata*. As a result, the primary marker compounds were identified with the aid of TOF-MS and MS/MS analyses. Compared with the raw material of *G. elata* with GEP5D, three new parishin-type compounds were identified according to their retention time, accurate mass, and fragmentation patterns. The chromatographic peak areas for marker compounds, including *S*-(gastrodin)-glutathione, *S*-(4-hydroxybenzylamine)-glutathione, and parishin-type compounds, changed significantly. This result indicated that by applying the “Jianchang Bang” method, changes in chemical composition in *G. elata* contents were observed. The study also demonstrated that chemometric analysis is helpful in understanding the processing mechanism and will provide scientific support for the clinical application of *G. elata*.

## 1. Introduction

The dry tuber of *G. elata*, commonly called as Tianma, Chi Jian, or Ming Tianma, has been officially listed in the Chinese Pharmacopeia [1] to treat symptoms of hyperactive liver, arrest endogenous wind, and stop tetany according to the theory of traditional Chinese medicine (TCM). *G. elata* is used for the prevention and treatment of neuralgic and nervous diseases, such as headaches, migraine, dizziness, rheumatism, convulsion, and epilepsy, for centuries in TCM [2–5]. Natural products have been widely accepted as important sources for nutritional supplements, dietary products, and drug discoveries due to their remarkable and multiple medical benefits for human health. On this account, herbal plants and their bioactive ingredients, especially those with new compounds, have been studied to highlight some of their potential therapeutic values and subjected to extensive research.

*G. elata* contains a variety of components that have been previously reported [6–8]. These structurally identified compounds were derived from organic solvent extracts of the rhizome which were produced from either fresh raw materials or from steamed and dried materials using different analytical techniques. In recent years, pharmaceutical studies suggested that phenolic glucoside gastrodin, gastrodigenin (*p*-hydroxybenzylalcohol), and parishins were identified as secondary metabolites responsible for biological activities [9, 10]. Other compounds with relatively low concentrations, such as nucleosides, polysaccharides, and phospholipids, have also been reported in the literature [11, 12]. The bioactive compounds in *G. elata* have increasingly become the focus of this research for important potential sources in developing new drug products [13].

*G. elata* has to be processed before clinical application according to the Chinese pharmacopeia. The processing of *G. elata* can cause changes in the content, potentially in the chemical property of the ingredients, or both of them, and influence its clinical efficacy. Processing methods, including mixing *G. elata* with wine, honey, *Tribulus terrestris*, wheat bran, or serofluid, have been recorded in some ancient books of TCM [14–17], but the most commonly used processing method is to steam and dry the tuber and then cut it into slices [18]. Among the main genres of TCM, “Jianchang Bang” is a genre located in the Nancheng county of Jiangxi Province in China. In this, steamed and sliced *G. elata* is mixed with ginger juice and then dried at room temperature. The processing method has been believed to be a special traditional processing means because of local clinical application of the theory of “toxicity reducing and efficacy enhancing.” After processing with ginger juice, the plain property of *G. elata* would be turned to warm, according to the theory of TCM. Compared with the pharmacopeia processing method, the product processed with ginger juice by the “Jianchang Bang” method might have more power to relieve headache and reduce side effects of vomiting caused by the smell of *G. elata*. Although the processed product of *G. elata* with ginger has shown good efficacy practically, there is limitation for its clinical application owing to the lack of scientific evidences. Therefore, finding out the differences in the chemical composition of *G. elata* obtained before and after processing by the “Jianchang Bang” method is the key to revealing the concoction mechanism that can be used for providing clinical application.

Many studies have been carried out and focused on the differences in composition changes in processed *G. elata* products [19–22]. Li et al. used the UHPLC/Q-TOF-MS/MS method for the identification of 64 chemical components in *G. elata* by comparing the retention time, accurate mass measurements, and characteristic fragmentation patterns [23]. Yet, these studies were only based on the conventional processing methods, such as steaming and drying or sulfur fumigation, rather than processing *G. elata* with accessories including ginger or wine. It is hard to determine and characterize the key chemical constituents and markers in processed *G. elata* using the “Jianchang Bang” method.

In order to understand the chemical composition of *G. elata* processed with ginger juice, a UHPLC-MS/MS combined with multivariate statistical analysis method was developed. The method facilitated the elucidation of characteristic fragmentation pathways of targeted and nontargeted constituents, especially of key chemical ones, in processed *G. elata* using the “Jianchang Bang” method by offering accurate mass measurement and significant molecular ions with different ionization modes, and then confirmed their molecular structures using the MS/MS fragmentation pattern. This analysis can also offer an excellent resolution of chromatographic separation of main components from different processed products of *G. elata*. This is the first time that UHPLC-MS/MS and chemometric methods are being used for the identification of *G. elata* processed with ginger using the “Jianchang Bang” method.

## 2. Materials and Methods

### 2.1. Chemicals and Reagents

Methanol, acetonitrile, and formic acid were of HPLC grade and purchased from Fisher Scientific (Fair Lawn, NJ, USA). Water used as the UHPLC mobile phase was purified using a Millipore Synergy UV Water Purification System (Millipore SAS, Molsheim, France). Reference standards parishin A (No: wkq17110609), parishin B (No: wkq17052501), and parishin C (No: wkq17120605) were purchased from Sichuan Vikki Biotechnology Co., Ltd. (Xi'an, China), and their purities were ≥98%.

Dried rhizomes of *G. elata* were obtained from Zhaotong county, Yunnan Province, China (Jiangxi Wuzhou Pharmaceutical Co., Ltd.) and identified by Dr. Lingyun Zhong (Jiangxi University of Traditional Chinese Medicine). Fresh ginger was purchased from a local supermarket in Mississippi, USA.

### 2.2. Sample Preparations

#### 2.2.1. Raw and Processed Plant Samples

*G. elata* (400 g) was steamed, cut into slices, and dried. Ginger juice (50 mL) was obtained by grinding fresh ginger using a mortar and pestle. Dried slices of *G. elata* (30 g) were mixed with the ginger juice (3.75 mL), which is the “Jianchang Bang” method. Three different materials, such as the raw material, dried slices of *G. elata* after steaming, and the one processed by the “Jianchang Bang” method (steamed and dried slices of *G. elata* processed in ginger juice for 5 days) were frozen at −20°C for a day and then taken out for freeze-drying (LABCONCO).

#### 2.2.2. Sample Preparation

After grinding to a fine powder using a pulverizing machine (Chart 5 Retsch. MM400), each sample (2.0 g), such as the raw material of *G. elata*, dried slices of *G. elata* after steaming, and samples processed by the “Jianchang Bang” method, was weighed in a centrifuge tube, then 3.5 mL of 50% methanol (methanol/water = 1 : 1, v/v) was added, and the mixture was sonicated in a water bath for 30 min. After that, the sample was centrifuged for 10 min at 959 ×*g*. The supernatant was transferred to a 10-ml volumetric flask. The procedure was repeated for a total of three times and the respective supernatants were combined. The final volume was adjusted to 10 ml with 50% methanol and mixed thoroughly. Prior to injection, an adequate sample (ca. 2 ml) was passed through a 0.45-*μ*m PTFE membrane filter and collected in an LC vial.

### 2.3. UHPLC Chromatographic Conditions

The UHPLC analyses were performed on a Waters Acquity UPLC™ system (Waters Corp., Milford, MA, USA) that included a binary solvent manager, a sample manager, a heated column compartment, and a photodiode array (PDA) detector [24]. The separation was carried out on an Acquity UPLC™ HSS T3 column (100 mm × 2.1 mm i.d., 1.8 *μ*m). The sample temperature and column temperature were maintained at 10°C and 45°C, respectively. The mobile phase consisted of water containing 0.05% formic acid (v/v) (A) and acetonitrile with 0.05% formic acid (B). The analysis was conducted at a flow rate of 0.5 mL/min and performed using the gradient elution as follows: 0–2.5 min, 1% B; 2.5–10 min, 1% B to 9% B; 10–14 min, 9% B to 20% B; 14–18 min, 20% B to 30% B; 18–21 min, 30% B to 60% B; 21–24 min, 60% B to 80% B; and 24–25 min, 80% B to 100% B. Each run was followed by a 3-min wash with 100% B and an equilibration period of 3.5 min with initial conditions. A Strong needle wash solution (90/10; acetonitrile/water, v/v) and a weak needle wash solution (10/90; acetonitrile/water, v/v) were used.

### 2.4. ESI-QTOF-MS

The high-resolution mass spectrometric analyses were performed using electrospray ionization (ESI) in the negative mode on a Waters Xevo G2-S QTOF mass spectrometer (Waters Corporation). The MS instrument was operated in the following conditions: mass scan range of 100–1500 Da, capillary voltage of 2.0 kV, cone voltage of 40 V, source temperature of 80°C, desolvation temperature of 450°C, desolvation gas flow of 900 L/hr, cone gas flow of 50 L/hr, and collision energy of 6 eV. Leucine-enkephalin was used for lock mass correction at a concentration of 5 *μ*g/mL and a flow rate of 10 *μ*L/min. Ions [M-H]^−^ (*m*/*z* 554.2615 Da) and fragments at *m*/*z* 276.6268 Da of leucine-enkephalin were applied to ensure mass accuracy during the MS analysis. The lock spray interval was set at 30 s, and the data were averaged over three scans. The mass spectrometer was programmed to switch between two steps: between low (10 V) and elevated (30–50 V) collision energies on the gas cell, using a scan time of 0.1 s per function.

### 2.5. Statistical Analysis

**Figure 1:**
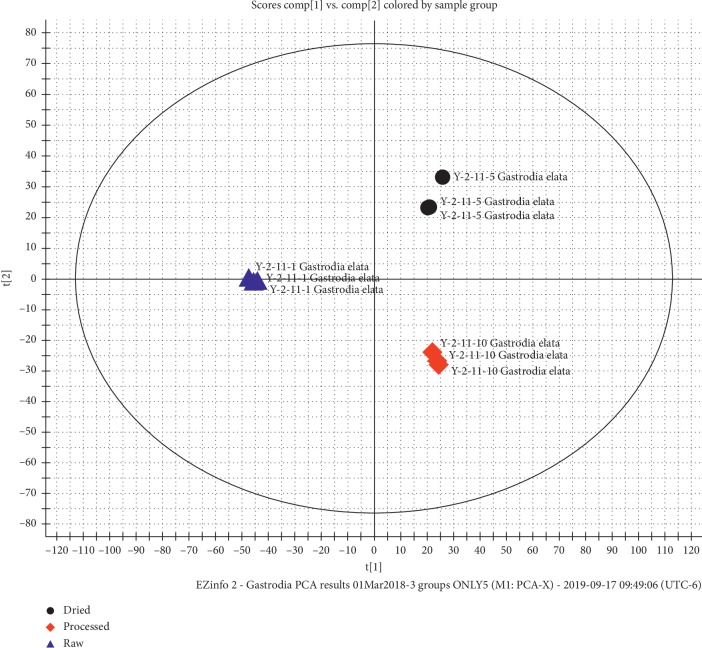
PCA results of *G. elata* raw material, S&D *G. elata*, and GEP5D samples.

PCA is one of the statistical analysis techniques used for finding the difference between different sample groups [25, 26]. In the study, data were achieved using Waters MassLynx™ Software v. 4.1 connected to MarkerLynx™ XS Application Manager (Waters Corporation, Milford, USA). A series of procedures in the software were used to handle a retention time of 0.1 min, the chromatogram from 0.2 to 25 min, the mass range of 100–1500 Da, the mass window of 0.02 Da, and the noise elimination level of 10.00%. All compounds identified in *G. elata* samples before and after processing using ginger juice were distinguished via PCA and Orthogonal Partial Least Squares-Discriminant Analysis (OPLS-DA) through the MarkerLynx XS Application Manager, as shown in Figure 1. The model has 3 score components for the samples: *G. elata* raw material, dried *G. elata*, and *G. elata* processed with GEP5D. These scores display a good separation for the three groups. The scores t[1] (*x*-axis) and t[2] (*y*-axis) are key variables in summarizing and separating the data. Each point in the plot corresponds to an observation. The groups are shown in different colors. This variation was assuredly caused by the use of the “Jiangchang Bang” method on *G. elata* in this study.

## 3. Results

**Figure 2:**
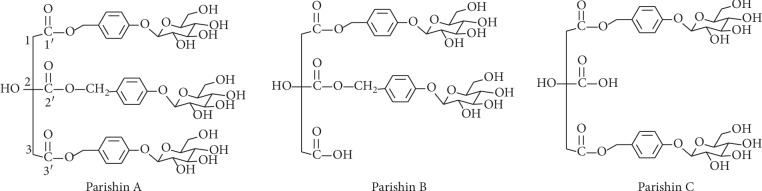
Structures of the compounds.

**Figure 3:**
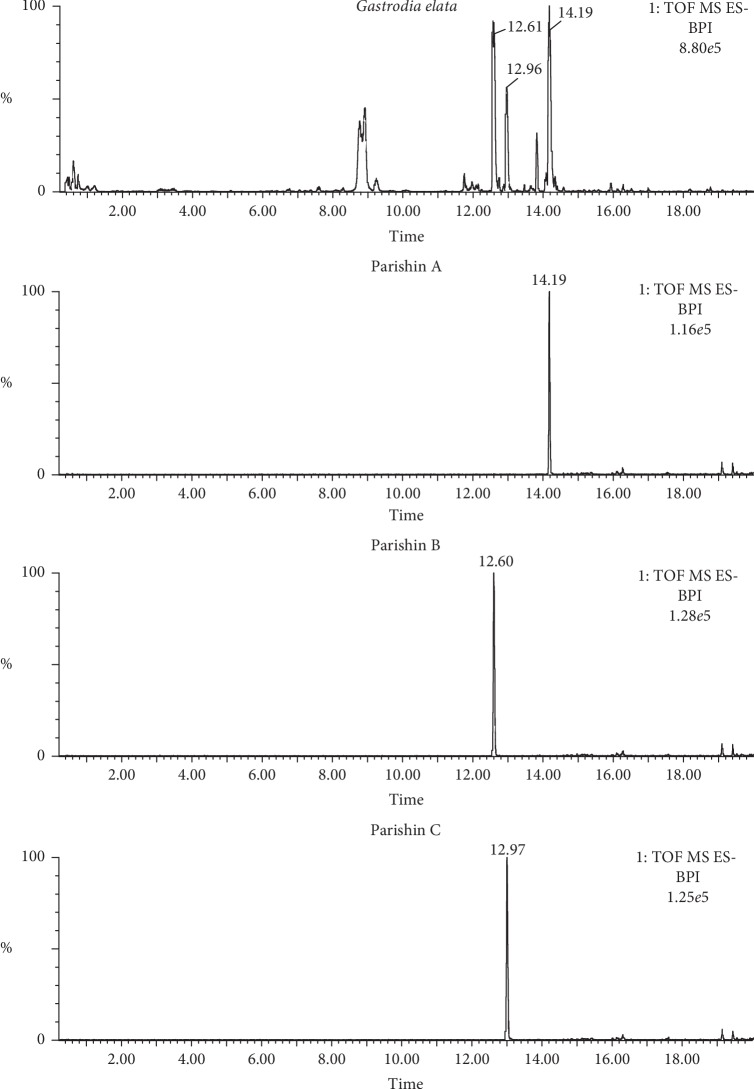
Chromatograms of *G. elata* and reference standards parishin A, B, and C.

**Figure 4:**
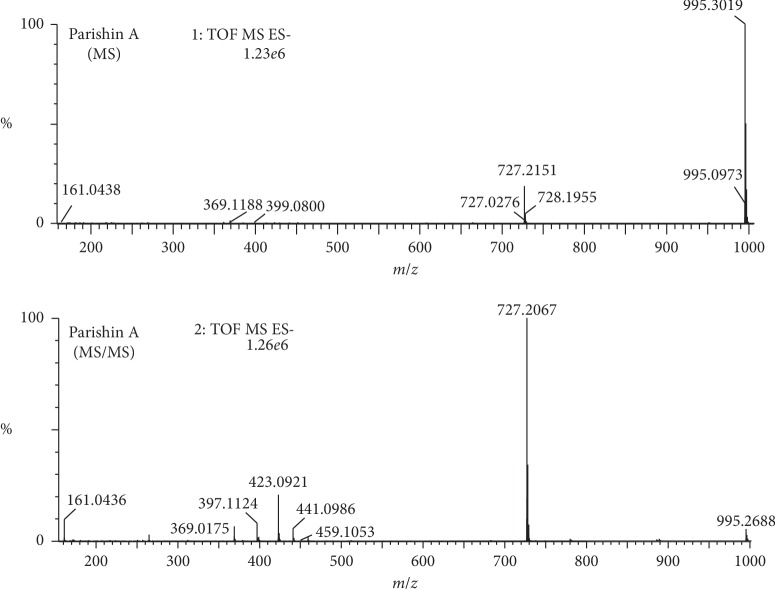
MS and MS/MS spectra of parishin A.

**Figure 5:**
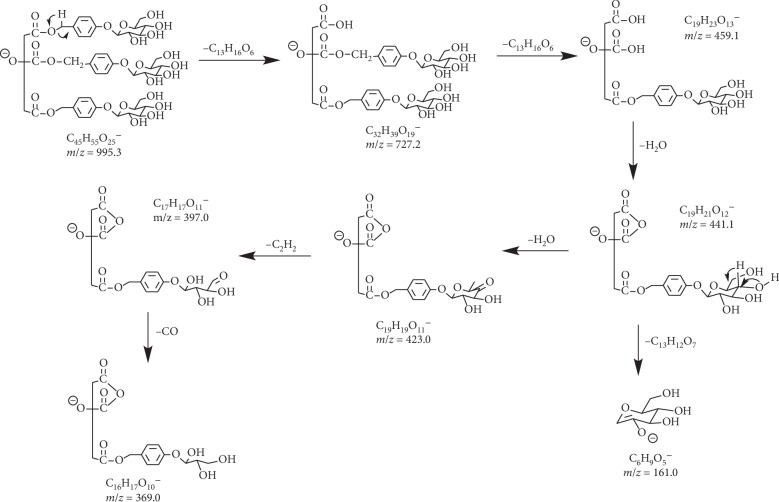
Proposed fragmentation pathway for parishin A.

Parishin A, B, and C (Figure 2) are major components in *G. elata* and eluted at 14.19, 12.64, and 13.01 min, respectively, under optimized conditions (Figure 3). Their structures in *G. elata* were unambiguously confirmed by comparing the retention time, accurate mass, and fragmentation pattern with that of reference standards. For instance, parishin A is a conjugate comprising three gastrodin units based on a citric acid unit by ester linkage. On the MS spectra of parishin A, ions at *m*/*z* 995.2997 Da are corresponding to the deprotonated molecular ion C_45_H_55_O_25_^−^ ([M-H]^−^, *calc*. 995.3038). When applying collision energy, it produces fragments at *m*/*z* 727.2067, 459.1053, 441.0986, 423.0921, 397.1124, 369.0175, and 161.0436 Da, corresponding to [M-C_13_H_16_O_6_]^−^, [727-C_13_H_16_O_6_]^−^, [459-H_2_O]^−^, [441-H_2_O]^−^, [423-C_2_H_4_]^−^, [397-CO]^−^, and [C_6_H_9_O_5_]^−^, respectively, that were observed in the MS/MS spectra of parishin A (Figure 4). The fragmentation pathway is proposed in Figure 5.

Parishin B and C are isomers that the gastrodin unit connects at 2′ and 3′, respectively (Figure 2). They showed different retention times but had fragmentation pattern identical to that of parishin A.

**Figure 6:**
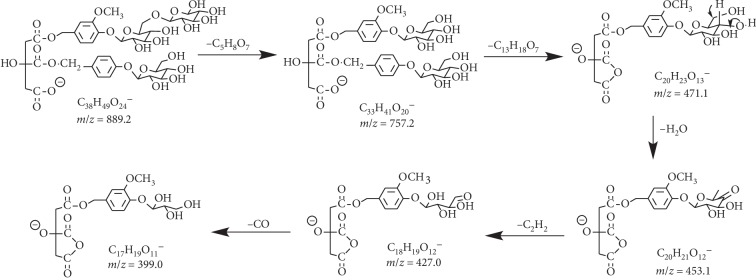
Proposed fragmentation pathway for compound **9**.

Compound **9** is eluted at 12.92 min and found to be a deprotonated molecular ion at 889.2624 Da ([C_38_H_49_O_24_]^−^, *calc*. 889.2614). When applying collision energy, it generates fragments at *m*/*z* 757.2218 Da [C_33_H_41_O_20_]^−^ corresponding to the loss of 132 Da. This indicates that compound **9** contains a pentose. Meanwhile, fragments at *m*/*z* 757 Da are 30 Da higher than key fragments at *m*/*z* 727 Da in parishin A, B, or C, which suggests that the fragment at *m*/*z* 757 Da exists as a methoxy group. In *G. elata*, reported compounds parishin L and H contain a methoxy unit on their molecules [23]. Therefore, the fragmentation pattern of compound **9** is proposed in Figure 6. This compound is identified as a pentose derivative of parishin H or its isomer, a new marker compound identified only in the processed sample by the “Jianchang Bang” method.

**Figure 7:**
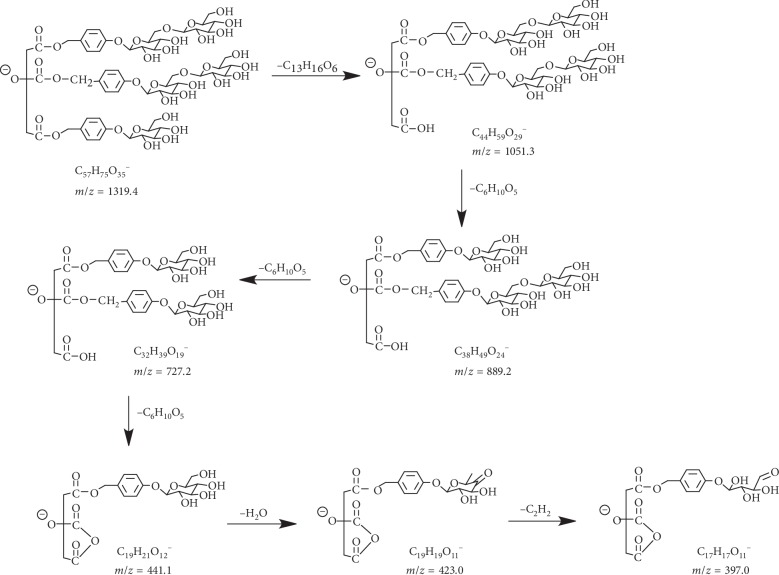
Proposed fragmentation pathway for compound **16**.

Compound **16** is a new mark in GEP5D that eluted at 13.9 min and appeared to be deprotonated molecular ion at 1319.4051 Da ([C_57_H_75_O_35_]^−^, *calc*. 1319.4094) but does not show in the raw sample at the same retention time. When employing collision energy, fragment ions at *m*/*z* 1051.3173 Da and 889.2628 Da related to the losses of gastrodin residue (268 Da) and hexose (162 Da), respectively, are observed. In the following fragmentation pathway, fragment ions of compound **16** are the same as that of parishin A. Therefore, compound **16** is tentatively identified as a derivative of parishin A plus two units of hexoses, according to its fragmentation pattern (Figure 7).

Deprotonated molecular ions of compound **14** are at 1157.3589 Da ([C_51_H_65_O_30_]^−^, *calc*. 1157.3566). The elution time of compound **14** (13.77 min) is different from that of parishin F (14.13 min) in *G. elata* samples, but compound **14** has a fragmentation pattern identical to that of parishin F [23]. Therefore, compound **14** is confirmed as an isomer of parishin F, a new marker compound found only in the GEP5D sample by the “Jianchang Bang” method, as shown in Table 1.

Peak areas of many key marker compounds in Table 1 are different in raw, S&D, and GEP5D samples. These compounds are mainly parishin-type compounds, except for compounds **1** and **5** which are derivatives of glutathione. As seen in Table 1, the peak areas of maker compounds in S&D samples are usually decreased when compared with that of raw samples. Among them, the most obvious decline is found for compound **12**: the peak area of this compound in the raw sample is more than sixfold that of the dried sample at the same optimized analysis conditions. The peak area of compound **3** also experiences a great decrease after processing *G. elata* by the drying method. Yet, there are few marker compounds, such as compounds **5**, **8**, and **11,** whose peak areas increase slightly when compared with raw samples and dried samples. However, the peak areas of some compounds, such as compounds **1**, **2**, **3**, **12**, **15**, and **19**, in GEP5D samples have been found to be lower than that of raw samples but higher than that of S&D samples. Moreover, the peak areas of compounds **6** and **11** in GEP5D samples are greater than that of raw samples. On the basis of MS and MS/MS data, it has been found that pentose or hexose derivatives of parishin-type compounds can be accumulated in *G. elata* samples processed by the “Jianchang Bang.” This observation has been proved by the identification of new marker compounds **9**, **14**, and **16**.

**Figure 8:**
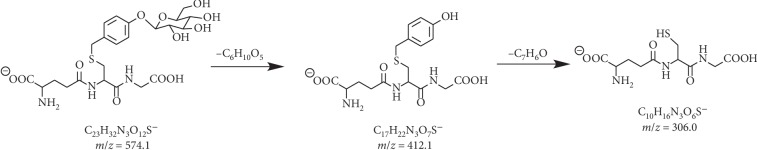
Proposed fragmentation pathway for compound **1**.

In Table 1, parishin-type compounds are characterized following the fragmentation pattern of parishin A, B, and C according to MS and MS/MS data. Two derivatives of glutathione are determined as follows. Deprotonated molecular ions of compound **5** at 12.32 min are at 412.1171 Da ([C_17_H_22_N_3_O_7_S]^−^, *calc*. 412.1184). On the MS/MS spectrum, the fragment ions at 306.0758 Da [C_10_H_16_N_3_O_6_S]^−^ correspond to the loss of the 4-hydroxy benzyl group. Therefore, compound **5** is tentatively identified as S-(4-hydroxybenzyl)-glutathione [25]. Compound **1** at 9.37 min (*m*/*z* 574.1700, [C_23_H_32_N_3_O_12_S]^−^, *calc*. 574.1712) is 162 Da higher than compound **5**, which suggests that compound **1** is a hexose derivative of compound **5**. Considering gastrodin as one of the main components, compound **1** is characterized as S-gastrodin glutathione, and its fragmentation pathway is shown in Figure 8.

## 4. Discussion

For the UHPLC/Q-TOF-MS/MS analysis, different UHPLC columns, column temperatures, mobile phases, gradient elutions, and flow rates were evaluated to achieve the optimal separation. As a result, acetonitrile–water with 0.05% formic acid combined with the optimized gradient elution on an UPLC HSS T3 column at 45°C offered an appropriate chromatographic separation and MS responses in the negative ion scan mode.

The OPLS-DA was used for the identification and differentiation of potential markers based on their peak areas [27]. As the most important observation, three new markers, such as compounds **9**, **14**, and **16**, along with at least 17 known compounds in *G. elata* were identified by the differences in compounds obtained from raw and GEP5D samples. In total, 20 key marker compounds were obtained from raw, S&D, and GEP5D samples based on differences identified by the comparison of their peak areas under the same optimized analysis conditions (Table 1).

An UHPLC-MS/MS method combined with multivariate statistical analysis was conducted to determine and characterize key marker compounds in *G. elata* raw material and processed samples by steaming or the “Jianchang Bang” method. Because of the significant evaluating means which reflected the changes in major compounds in different samples of *G. elata* processed by ginger, new marker compounds have been found and many important constituents experienced great changes between raw and GEP5D or raw and dried samples in peak areas.

## 5. Conclusions

In this study, unsupervised PCA can differentiate *G. elata* raw material, steamed samples, and samples processed by the “Jianchang Bang” method. OPLS-DA of raw material vs. GEP5D samples processed by the “Jianchang Bang” method provides a tool to select and determine marker compounds. A total of 20 marker compounds were characterized based on the accurate mass of MS and fragment ions of MS/MS data as well as by comparing with corresponding reference standards and literature. Compounds **9**, **14**, and **16** are new markers of parishin-type compounds in *G. elata* samples processed with ginger by the “Jianchang Bang” method. Except for two derivatives of glutathione (**1** and **5**), other marker compounds are parishin-type constituents. These results suggest that the developed UHPLC-MS/MS method combined with PCA and OPLS-DA can characterize key components in *G. elata* processed with ginger. This method will support the clinical application of *G. elata* processed by the “Jianchang Bang” method in a scientific way.

## Figures and Tables

**Table 1:** Key marker compounds in *G. elata* raw material, steamed and dried, and GEP5D samples.

Compound	Retention time (min)	Mass	Peak area			Name	Fragmentation ions
			Raw	S&D	GEP5D		
1	9.37	574.1700	13383	3303	4978	S-(Gastrodin)-glutathione	412.1168, 306.0758, 272.0877, 254.0773, 210.0867
2	11.80	727.2089	7734	2661	2835	Isomer of parishin B	441.1028, 397.1129, 369.1189, 161.0447
3	12.02	727.2082	6542	1301	2613	Isomer of parishin B	441.1017, 423.0917, 399.0924, 397.1118, 369.1176, 161.0438
4	12.20	889.2631	5478	4481	3680	Isomer of parishin H	727.2081, 441.1037, 423.0923, 397.1158, 323.0960, 263.0748, 161.0435
5	12.32	412.1171	1055	1530	997	*S*-(4-Hydroxybenzyl)-glutathione	306.0733, 272.0825, 254.0826, 210.0857
6	12.57	889.2625	765	221	2663	Isomer of parishin V	727.2065, 423.0901, 323.0976, 263.0471, 161.0442
7	12.64	727.2089	111564	95596	92543	Parishin B	441.1028, 423.0928, 397.1134, 263.0757, 161.0419
8	12.78	889.2626	6887	9974	6442	Parshin V	727.2051, 423.0912, 397.1130, 323.0970, 161.0440
9	12.92	889.2624	0	0	917	Pentose derivative of isomer of parishin H	757.2218, 423.0952, 397.1127, 323.0952, 161.0444
10	13.01	727.2086	54794	50144	44433	Parishin C	441.1022, 423.0923, 397.1125, 369.1179, 161.0443
11	13.12	889.2621	1681	3005	1731	Parishin I	727.2092, 423.0890, 323.0974, 161.0443
12	13.51	473.1298	1874	307	402	Demthyl derivative of parishin N	441.1042, 423.0969, 397.1045, 169.0110
13	13.69	1157.3590	5981	1816	1638	Isomer of parishin F	889.2646, 727.2120, 423.0960, 323.1023, 161.0433
14	13.77	1157.3589	0	216	562	Isomer of parishin F	889.2661, 727.2122, 423.0913, 323.0916, 161.0523
15	13.86	473.1290	6617	1399	2181	Demthyl derivative of parishin N	423.0891, 397.1096, 169.0130
16	13.90	1319.4051	0	14	590	Derivative obtained by adding two glucose to parishin A	1051.3173, 889.2628, 727.2076, 473.1293, 423.0913, 397.1132, 169.0135
17	14.13	1157.3595	10521	6599	7572	Parishin F	889.2638, 727.2078, 423.0935, 323.0958, 161.0448
18	14.19	995.3057	117584	89968	87400	Parishin A	727.2077, 441.1008, 423.0918, 397.1118, 369.1174, 161.0446
19	14.21	727.2092	25233	16420	17690	Isomer of parishin B	441.1025, 423.0920, 397.1129, 369.1180, 161.0401
20	14.37	1025.3162	7106	4154	3181	Parishin L	889.2578, 757.2204, 727.2094, 423.0911, 369.1133, 161.0443

## Data Availability

The data used to support the findings of this study are included within the article.

## Abbreviations

*G. elata*: *Gastrodia elata*
TCM: Traditional Chinese medicine
S&D: Steamed and dried
GEP5D: *G. elata* was processed with ginger juice for up to 5 days
UHPLC-MS/MS: Ultra-high-performance liquid chromatography-tandem mass spectrometry
PCA: Principal component analysis
OPLS-DA: Orthogonal partial least squares-discriminant analysis
TOF: Time of flight
ESI: Electrospray ionization
ESI-QTOF-MS: Electrospray ionization quadrupole time-of-flight mass spectrometry
UHPLC/Q-TOF-MS/MS: Ultra-high-performance liquid chromatography-quadrupole time-of-flight tandem mass spectrometry.
